# Nonadherence to Diabetes Complications Screening in a Multiethnic Asian Population: Protocol for a Mixed Methods Prospective Study

**DOI:** 10.2196/63253

**Published:** 2025-05-08

**Authors:** Amudha Aravindhan, Eva Fenwick, Aurora Wing Dan Chan, Ryan Eyn Kidd Man, Wern Ee Tang, Ngiap Chuan Tan, Charumathi Sabanayagam, Junxing Chay, Lok Pui Ng, Wei Teen Wong, Wern Fern Soo, Shin Wei Lim, Ecosse L Lamoureux

**Affiliations:** 1 Singapore Eye Research Institute Singapore National Eye Centre Singapore Singapore; 2 Duke-NUS Medical School Singapore Singapore; 3 National Healthcare Group Polyclinics Singapore Singapore; 4 SingHealth Polyclinics Singapore Singapore; 5 The University of Melbourne Melbourne Australia

**Keywords:** diabetes complications screening, diabetic retinopathy, diabetic nephropathy, diabetic foot complications, nonadherence, mixed methods, microvascular complications, quality of life

## Abstract

**Background:**

Yearly screening for microvascular complications of diabetes mellitus (DM), namely diabetic retinopathy (DR), diabetic nephropathy (DN), and diabetic foot complications (DFC), is recommended to reduce their incidence, and delay or prevent their progression. Poor adherence to screening is common, but prospective data on the magnitude and predictors of nonadherence to DR, DN, and DFC screening in Singapore are unavailable.

**Objective:**

The Understanding Non-Adherence to Diabetes Complications Screening study aims to determine the rates, predictors, and clinical and economic impact of nonadherence to diabetic complications screening in patients with type 2 diabetes in Singapore. The study describes the methodology and participants’ baseline characteristics that may be associated with nonadherence to DM complications screening.

**Methods:**

In this prospective, mixed methods, clinic-based study, patients who underwent DR, DN, or DFC screening and were offered an annual rescreening appointment, were recruited from 6 primary care centers. Patients’ sociodemographic, medical, clinical, and patient-reported characteristics were recorded at baseline. Nonadherence to DR, DN, or DFC screening is defined as not attending the annual rescreening appointment within 4 months of the scheduled rescreening date. Adherence and clinical data will be recorded at 16 months post enrollment. Additionally, selected participants and health care professionals will undergo qualitative interviews to elicit barriers or facilitators of adherence to rescreening.

**Results:**

Ethical approval was obtained in November 2016. Study enrollment commenced across the 6 sites between June 2018 and February 2019, and baseline data collection ended at all sites in January 2020. 974 eligible patients (2123 screenings; median age of 61.0, IQR 55.0-67.0 years; male: 515, 52.9%; Chinese: 624, 64.1%) consented and completed the baseline assessment. Of these, 734 (75.4%), 603 (61.9%), and 786 (80.7%) attended DR, DN, and DFC screening, respectively. Most (n=793, 81.4%) attended more than 1 complication screening on the same day; had received secondary or lower education (n=701, 71.9%); had hypertension (n=711, 73.4%) and dyslipidemia (n=828, 85.1%); and 43.1% (n=419) were obese (BMI>27.5 kg/m^2^). Median DM duration and hemoglobin A_1c_ levels were 6.3 (IQR 3.0-12.0) years and 6.9% (6.4%-7.6%), respectively. Over half (n=532, 55.1%) had not received prior DM education. Furthermore, participants reported low levels of diabetes-related self-efficacy (median 1.4, IQR 1.0-3.9 out of 5).

**Conclusions:**

At baseline, we have successfully enrolled almost 1000 patients with type 2 diabetes scheduled for annual DR, DN, or DFC rescreening, and potential predictors of nonadherence to rescreening were systematically collected. Follow-up phases will focus on establishing the rates and associated modifiable predictors of nonadherence to DR, DN, or DFC rescreening, which may inform program initiatives.

**International Registered Report Identifier (IRRID):**

DERR1-10.2196/63253

## Introduction

Microvascular complications of diabetes mellitus (DM), namely diabetic retinopathy (DR), diabetic nephropathy (DN), and diabetic foot complications (DFC) are common, and impose a profound quality of life, health, and economic burden on the individual and society [1,2]. Regular screening for these complications can reduce their incidence and delay or prevent the progression of blindness, end-stage renal disease, and diabetic foot ulcers [3-7]. In Singapore, guidelines recommend that individuals with DM have a dilated fundus examination, assessment of urine albumin excretion and serum creatinine, and foot examination or education annually. However, screening programs are only cost- and clinically-effective if uptake is high, and studies conducted overseas have shown poor adherence to screening, ranging from 20% to 60% [8-10]. At a population-based level, the Singapore Epidemiology of Eye Disease study (2004-2011) showed that 80% of individuals with DM had undiagnosed DR, a proxy indicator of nonadherence to DR screening [11]. However, current data on nonadherence to DR, DN, and DFC screening in Singapore are lacking.

Several factors have been associated with nonadherence to DM complications screening including low educational attainment, issues with appointment access, and lack of social or family support [8,12-17]. However, most existing research is limited by a retrospective study design, or only using qualitative data collection, meaning that predictors of nonadherence to DM complications screening remain largely unknown. Additionally, nonadherence to screening has been associated with poor DM control [6,18], more severe disease [19,20], frequent treatment [21], and hospitalization needs [6]. Indeed, a systematic review exploring patients’ nonattendance at eye screening programs revealed that repeated nonattendance was associated with an increased risk of sight-threatening DR [20]. However, the studies included were conducted mostly in Western populations and may not be applicable to Asian populations due to different screening guidelines, and regional, political, social, economic, cultural, and health care systems.

To date, there are no prospective and contemporary population data on the rates; predictors; and clinical and economic impact of nonadherence to DM complications screening in Singapore. Furthermore, our understanding of the barriers or facilitators to screening adherence faced by patients with DM is limited. Such information is crucial for health care professionals and policy makers to make decisions on resource allocation and for informing interventions to improve adherence to DM screening programs. Against this background, we are conducting a prospective, multicentered clinic-based study (Understanding Non-Adherence to Diabetes Complications Screening [UNADS]) to ascertain the rates (aim 1); associated predictors (aims 2a and 2b); and clinical (aim 3) and economic impact (aim 4) of nonadherence to DM complications screening in primary care “polyclinic” patients with type 2 DM (T2DM) in Singapore using a mixed methods approach. In this paper, we provide a detailed description of the study design, data collection tools and protocols, and associated analytical plans. Furthermore, we present the patients’ baseline characteristics and suggest factors that may potentially be associated with nonadherence to DM complications screening.

## Methods

### Conceptual Framework

We used Green and Kreuter Predisposing, Reinforcing, and Enabling Constructs in Educational Diagnosis and Evaluation (PRECEDE) and Policy, Regulatory, and Organizational Constructs in Educational and Environmental Development (PROCEED) model [22] to inform our mixed methods study design. This model has two distinct components: (1) “PRECEDE” which constitutes five diagnostic and planning phases to facilitate the identification of priorities and setting of objectives, and (2) PROCEED which consists of four phases and focuses on the identification of criteria for policy implementation and subsequent evaluation. PRECEDE-PROCEED has been widely used to identify and conceptualize barriers to health care screening in other health fields [23-26]. Our study used the first 5 “PRECEDE” phases (Figure S1 in Multimedia Appendix 1). The first phase (social diagnosis) establishes the health problems which impact the quality of life of the target population and the second phase (epidemiological diagnosis) documents the importance of these problems through survey and epidemiological data. The third phase (behavioral and environmental diagnosis) focuses on the systematic identification of health practices and other factors linked to health problems. The fourth phase (educational and ecological diagnosis) identifies the factors that, when modified, will most likely result in the desired behavior change. These factors are then classified as predisposing, enabling, or reinforcing. The last phase (administrative or policy assessment) of PRECEDE analyses policies, resources, and circumstances prevailing organizational situations that could hinder or facilitate meeting the objectives of the fourth phase. Based on our consultations with senior health care professionals from the SingHealth Polyclinics (SHP) and 2 National Healthcare Group Polyclinics (NHGP), and data from the NHG DM registry and Singapore Epidemiology of Eye Disease study, nonadherence to DM complications screening was already identified as a major public health issue in Singapore [11,27]. Therefore, in this study, we focus on the implementation of phases 2-5 via a prospective, mixed methods study that combines a quantitative data collection phase followed by in-depth qualitative interviews.

### Study Setting and Design

In Singapore, DM complications screening is performed mainly at 23 primary health care government polyclinics. Of these, 4 SHP and 2 NHGPs were involved in our study, based on high attendance by minority ethnic groups, that is, Malays (Geylang [NHGP]) and Indians (Hougang [NHGP]), and high patient volume to maximize recruitment potential (ie, Bedok, Bukit Merah, Outram, and Pasir Ris [SHP]).

**Figure 1:**
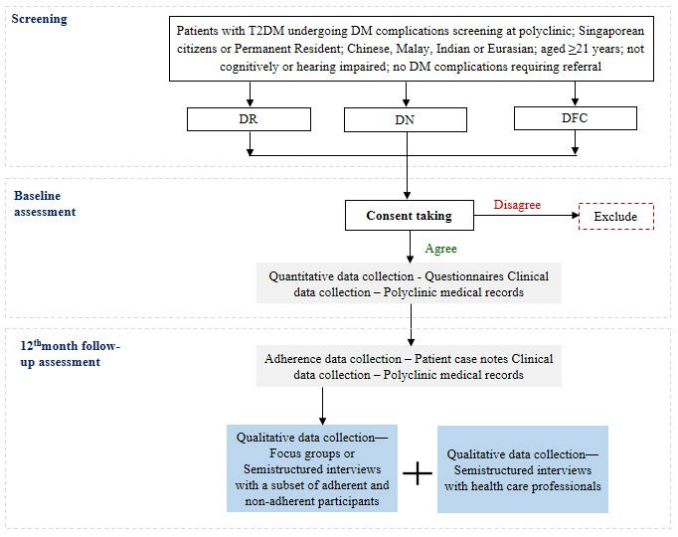
UNADS mixed methods study design. DFC: diabetic foot complication; DM: diabetes mellitus; DN: diabetic nephropathy; DR: diabetic retinopathy; T2DM: type-2 diabetes mellitus; UNADS: Understanding Non-Adherence to Diabetes Complications Screening.

Standardized and uniform research protocols were applied at all sites. Each participant completed a baseline assessment on the day of enrollment and will be followed up at 16 months post enrollment (Figure 1). Baseline assessments involved the collection of quantitative data including clinical and sociodemographic information, and patient-reported outcomes. At 16 months following the baseline assessment (ie, up to 4 months after the scheduled annual rescreen appointment), a trained clinical research coordinator will determine if the participant attended the annual rescreening appointment from polyclinic records. Participants who attended or did not attend rescreening will be deemed “adherent” or “nonadherent,” respectively. The clinical research coordinator will then arrange a follow-up visit for all participants (both adherent and nonadherent) at the study site to collect study-related clinical information that is not available in the electronic medical records (EMRs). Furthermore, purposively selected participants will be invited to participate in focus groups or semistructured interviews regarding barriers or facilitators to adherence with DM complications screening. In addition, semistructured interviews will also be conducted with health care professionals (eg, polyclinic doctors and allied health professionals) to elicit their perceptions on why some patients do not comply with their screening visits.

### Ethical Considerations

The study was approved by the SingHealth Centralized institutional review board (reference #2016/3041), and all patients enrolled in this study provided written informed consent prior to participation. All health care professionals who will be enrolled in qualitative interviews will be verbally consented prior to participation. The study was conducted in accordance with the Declaration of Helsinki.

To ensure confidentiality, each patient is assigned a unique identification number linked to the identifiable data (eg, name, contact details), which are saved separately on a password-protected system in a password-protected file, at each study site. Hard copies of the data were stored in secured cabinets at each study site. Audio files will be coded with the participant’s identification number but without any information that could be used to identify them. Audio files and transcripts were stored in password-protected folders in shared drives of the Singapore Eye Research Institute. All data collected, including transcripts of the audio files, will be only accessible to approved research team members.

Each study participant will receive a reimbursement of S $20 (US $15.29) for each screening type for which they are recruited as part of the study. For example, a participant who completes baseline assessments for attending DR and DN screening will receive a total of S $40 (US $30.57) worth of vouchers. Similarly, the participant will receive a total of S $40 (US $30.57) worth of vouchers upon completing the respective follow-up assessments.

### Study Population and Procedures

#### Quantitative Phase

##### Population

Patients with T2DM who underwent DM complications screening at one of the study sites and received a referral for an annual rescreen at the participating sites were eligible. All eligible participants were Singaporean citizens or permanent residents of Chinese, Malay, Indian, or Eurasian ethnicity; aged 21 years and older; free of significant hearing impairment that could affect the study protocol, and cognitive impairment assessed using the 6-item Cognitive Impairment Test [28]. Patients who had type 1 DM or with complications necessitating referral to tertiary care (eg, presence of moderate or worse DR, overt proteinuria, or neuropathy) were deemed ineligible to participate. Each patient was assigned a unique study identification number for each type of screening attended and enrolled in this study. For example, a patient attending DR and DN screening appointments on the same or different days (bundle screening) was assigned 2 unique study identification numbers.

##### Sample Size Determination

Assuming the nonadherence rate for DR, DN, and DFC is 50%, 30%, and 40%, a sample size of 1111 for DR, 933 for DN, and 1067 for DFC was needed to obtain a 95% CI of ±3% around the rates of nonadherence estimates with 80% power. Accounting for nonresponse and loss to follow-up of 20%, a sample size of 1444 for DR, 1213 for DN, and 1387 for DFC was needed. Furthermore, assuming a nonadherence rate of 50% and a probability of exposure (barriers or facilitators) of 15% among those who are adherent, these proposed sample sizes would be able to detect an odds ratio (OR) of 1.47, 1.6, and 1.5 of nonadherence to DR, DN, and DFC screening, respectively, for a barrier or facilitator. Additionally, assuming that the effect of bundle screening would be 30% on DN and DFC screening where DR screening (n=1444) was treated as a constant, we required 849 and 971 for DN and DFC, respectively. In total, we required a sample of 3264 patients.

##### Data Collection

All study assessments were undertaken by trained project members. A full description of all study procedures is provided in Table S1 in Multimedia Appendix 2.

- Sociodemographic, medical, and diabetes-related information and social history were collected via an in-house questionnaire and from EMR.
- Anthropometric parameters: height and weight were measured on the day of assessment if not available in the participant’s EMR.
- Clinical parameters: blood pressure (BP) was assessed using a digital automatic BP monitor [29] Dinamap model Pro Series DP110X-RW, 100V2; GE Medical Systems Information Technologies, Inc) on site if not available in EMR. Participants’ hemoglobin A1c (HbA1c), lipids (low- or high-density lipoprotein cholesterol, triglycerides, total cholesterol), serum creatinine, urine albumin creatinine ratio, and estimated glomerular filtration rate (eGFR) were collected from EMR if assessed 12 months or less. Otherwise, a venous blood sample drawn via venipuncture and a midstream urine sample were sent to Innoquest Laboratories Pte Ltd or National Healthcare Diagnostics, in Singapore for same-day analysis. The eGFR was calculated using the Chronic Kidney Disease Epidemiology Collaboration formula [30].
- Ocular parameters: retinal graded results of participants who attended the Singapore Integrated Diabetic Retinopathy Programme, a teleophthalmology-based screening program, were obtained from participants’ EMR.
- Patient-reported outcomes hypothesized to be associated with screening adherence were assessed using the following validated questionnaires: Revised Diabetes Knowledge Test [31,32], Health Literacy Test for Singapore questionnaire [33], 3-item Health Literacy Questionnaire [34], Diabetes Empowerment Scale (Short form)–Self Efficacy [35], Problem Areas in Diabetes Questionnaire [36], and Questions on Social Support [37,38].
- Specially crafted items designed to elicit barriers from the patient’s perspective based on known barriers from the literature and expert panel input were also included (eg, history of adherence or nonadherence to DM complications screening, time taken to reach screening appointments, DM-related education needs and experiences, and attitudes toward diabetes control).

All baseline quantitative data were collected in hard copy format and subsequently entered into the REDCap (Research Electronic Data Capture; Vanderbilt University) system.

##### Analytical Plan

Basic proportions will be used to assess the rate of nonadherence to each DM complications screening, and 2-screening bundles (eg, either DR+DN, DR+DFC, or DN+DFC screenings) and 3-screening bundles (ie, same-day screenings for all 3 complications; aim 1). Baseline sociodemographic, medical, clinical, and patient-reported factors will be analyzed continuously or categorically where appropriate, and logistic regression models will determine the associations between the various factors with nonadherence to each of the 3 DM complications screening visits, as well as to the 2- and 3- screening bundles (aim 2a). Linear and logistic regression models will be used to examine the relationships between nonadherence to each individual DM complications screening with intermediate pre-post changes in clinical outcomes collected at the follow-up visit (aim 3). To model the economic burden of nonadherence to DM screening, we will estimate and compare the cost of patients who are adherent versus nonadherent (aim 4). We will focus on long-term costs of nonadherence (time horizon: 40 years) as there is unlikely to be any substantial cost-saving in the short term. To estimate long-term costs of nonadherence, a decision tree model will be used, which captures possible benefits of screening adherence in terms of lower risk of developing complications and timely detection and treatment if complications occur which, in turn, would lead to lower costs in the long term. All analyses will be performed using Stata/SE (version 15; StataCorp).

#### Qualitative Phase

Our qualitative methodology aligns with the Consolidated Criteria for Reporting Qualitative Research (COREQ) checklist (Multimedia Appendix 3) [39].

#### Population

Focus groups or individual semistructured interviews will be conducted with both adherent and nonadherent participants for the 3 DM complications screenings. Participants will be purposively selected across the spectrum of age, sex, and language spoken (ie, English, Mandarin, Malay, and Tamil) to ensure the applicability of results. In addition, health care professionals, including advanced practice nurses, nurse care managers or educators, and family physicians, will be invited to participate in semistructured interviews.

#### Sample Size Estimation

Nonadherent participants will be oversampled to focus on barriers to screening adherence as these are amenable to intervention. Interviews will be offered in English, Mandarin, Malay, or Tamil to capture issues applicable to English or non-English speakers. We estimate that 8 focus groups (3 “adherent” and 5 “nonadherent”) with 5 to 7 participants per group, and 15-20 semistructured interviews will be required, resulting in a total of 55-76 participants. We estimate that 8-10 health care professionals will also be needed for the semistructured interviews. Sampling will continue until thematic saturation is reached (ie, no new themes emerge).

#### Data Collection

Patient focus groups and semistructured interviews will be conducted by trained interviewers and note takers face-to-face at the Singapore Eye Research Institute or over the phone, after the completion of the 16-month follow-up. Sessions will be conducted according to an interview guide developed from a thorough literature review and based on expert panel opinion. The interview guide will comprise open-ended questions targeting predisposing (eg, beliefs about illness and Western and Chinese medicine), reinforcing (eg, role of family members in DM care), and enabling (eg, importance of screening) factors guided by the PRECEDE component (see Multimedia Appendix 4 for the full patient interview guides). Different guides will be used for adherent and nonadherent participants.

We will use the Theoretical Domains Framework (TDF) to inform our interview guide for health care professionals and synthesize our qualitative data (see Multimedia Appendix 4 for full health care professional interview guides) [40]. The TDF covers 12 domains (eg, knowledge, skills, beliefs, and motivation) that cover potentially modifiable barriers that can inform intervention [41]. We will include open-ended questions like “What factors do you think patients consider when they are deciding whether or not to be screened?” as well as questions about knowledge of screening guidelines, referral practices, and information provided to patients regarding the referral during the consultation.

All qualitative interviews will be audio-recorded using a digital recorder. Audio files will be transcribed (and translated into English where necessary) by a professional transcription and translation company and will be checked by the research team for accuracy.

#### Analytical Plan

An inductive analytical approach will be adopted based on the constant comparative method whereby broad themes will be developed from the raw content of the transcripts [42]. New or improved themes that emerge from later transcripts will be incorporated into the coding hierarchy, and earlier transcripts will be updated to reflect these modifications. Themes will be categorized as predisposing, enabling, and reinforcing barriers or facilitators based on phase 4 of the PRECEDE component. From the health care professionals’ interviews, data relating to factors influencing behavior during complications referral will be coded using the 12 TDF domains. The computer program QSR NVivo 12 will be used to code the data. Transcripts will be analyzed by two different coders and disagreement in coding will be resolved by discussion or a third, independent coder (aim 2b).

#### Preliminary Analyses

In this preliminary report, we compared the characteristics of participants and those who refused to participate (nonparticipants) using 2-tailed *t* test or chi-square statistics, as appropriate. Furthermore, we present baseline characteristics that may be associated with nonadherence to DM complications rescreening using descriptive statistics (mean [SD], median [IQR], counts, and percentages). All preliminary analyses were performed using Stata/SE, version 15.

## Results

### Recruitment and Retention

Recruitment commenced on June 6, 2018, at Hougang June 11, 2018, at Bedok; June 19, 2018 at Outram; September 25, 2018, at Geylang; November 21, 2018, at Pasir Ris; and February 8, 2019, at Bukit Merah. The baseline quantitative data collection ended at all 6 sites on January 31, 2020.

A total of 3546 patients were screened across the 6 polyclinics. Of these, 830 (23.4%; Figure S2 in Multimedia Appendix 1) were excluded because (1) they received a less than 12-month (approximately 3-6 months) review referral at the polyclinic (n=604, 72.8%); (2) had significant hearing impairment (n=63, 8%); (3) had complications requiring referral to tertiary care (n=39, 5%); (4) had cognitive impairment (n=38, 5%); or (5) were not Chinese, Malay, Indian, or Eurasian (n=35, 4%). Of the remaining 2716 (76.6%) eligible patients, nearly two-thirds refused (n=1742, 64.1%) participation. Reasons for refusal included not being interested (n=901, 51.7%), lack of time (n=512, 29.4%), more than 1 study-related visit required (n=287, 16.5%), and “other” reasons (eg, does not want to share confidential information, the caregiver does not approve, does not believe in research; n=42, 2%). The remaining 974 (35.9%) eligible patients agreed to participate.

The response rate ranged from 9.5% in Bedok and 33.9% in PR to 85.0% in Geylang (Table 1). There were no significant differences in age and sex between participants and nonparticipants. Compared to participants, nonparticipants were more likely to only attend DR screening (n=730, 51.9%), not attend “bundle” screening appointments (n=909, 64.6%) and be from the Bedok site (n=498, 28.6%; all *P*<.001).

**Table 1:** Response rates of eligible patients to the UNADS^a^ study by polyclinic sites.

Polyclinic sites	Eligible patients, n	Participants, n	Response rate, %^b^
SHP^c^–Bedok	550	52	9.5
SHP–Outram	638	197	30.9
SHP–Pasir Ris	322	109	33.9
SHP–Bukit Merah	365	57	15.6
NHGP^d^–Hougang	494	264	53.4
NHGP–Geylang	347	295	85.0
Total	2716	974	35.9

^a^UNADS: Understanding Non-Adherence to Diabetes Complications Screening.

^b^Calculated as participants as a percentage of eligible patients.

^c^SHP: SingHealth Polyclinics.

^d^NHGP: National Healthcare Group Polyclinics.

### Baseline Profile of Study Participants

Of the 974 enrolled patients at baseline, 75.4% (n=734), 61.9% (n=603), and 80.7% (n=786) attended DR, DN, and DFC screening, respectively. Over 4 in 5 (n=793, 81.4%) attended 1 or more complication screenings on the day of enrollment.

Patient characteristics potentially associated with nonadherence to DM complications screening are outlined in the following sections.

### Sociodemographics

The baseline cohort had a median age of 61.0 (IQR 55.0-67.0) years, with over two-fifths less than 60 years (n=413, 42.4%); and were primarily male (n=515, 52.9%; Table 2). Most participants were Chinese (n=624, 64.1%), followed by Malay (n=208, 21.4%), Indian (n=136, 13.9%), and Eurasian (n=6, 1%). A majority had received secondary or lower education (n=701, 71.9%), were employed (n=589, 60.5%), had a monthly household income of more than S $2000 (n=518, 62.5%), and lived in public housing (n=843, 86.6%). Furthermore, most were married (n=737, 75.7%) and reported taking less than 30 minutes to access the polyclinic site (n=720, 74.3%).

**Table 2:** Baseline sociodemographic and lifestyle characteristics of UNADS^a^ study participants.

Characteristics					Values
**Screening attended, n (%)**					
	Diabetic retinopathy			734 (75.4)	
	Diabetic nephropathy			603 (61.9)	
	Diabetic foot complication			786 (80.7)	
Bundle screening on the same day, n (%)					793 (81.4)
**Sociodemographic factors**					
	**Age (years), median (IQR)**			61.0 (55.0-67.0)	
	**Age groups (years), n (%)**				
		≤49	118 (12.11)		
		50-59	295 (30.29)		
		60-69	420 (43.12)		
		>70	141 (14.48)		
	Sex, (male), n (%)			515 (52.9)	
	**Ethnicity, n (%)**				
		Chinese	624 (64.1)		
		Malay	208 (21.4)		
		Indian	136 (13.9)		
		Eurasian	6 (0.6)		
	**Educational attainment, n (%)**				
		Primary or lower	153 (15.7)		
		Secondary or A-level	548 (56.3)		
		Tertiary	273 (28)		
	**Marital status, n (%)**				
		Single	117 (12)		
		Married	737 (75.7)		
		Divorced or separated	61 (6.3)		
		Widowed	59 (6.1)		
	**Housing type**				
		Public	843 (86.6)		
		Private	131 (13.5)		
	**Monthly household income**				
		≤S $2000 (US $1524.80)	311 (37.5)		
		>S $2000	518 (62.5)		
	Employment status (yes), n (%)			589 (60.5)	
	**Accessibility to polyclinic**				
		Less than 30 minutes	720 (74.3)		
		30 minutes to 2 hours	249 (25.7)		
**Lifestyle or health behavior factors**					
	**Smoking**				
		Never or past smoker	870 (89.3)		
		Current smoker	104 (10.7)		
	**Alcohol status**				
		Never or past drinker	763 (78.3)		
		Current drinker	211 (21.7)		

^a^UNADS: Understanding Non-Adherence to Diabetes Complications Screening.

### Medical and Clinical

Comorbidities were common overall, with a high prevalence of hypertension (n=711, 73.4%), dyslipidemia (n=828, 85.1%), and obesity (n=419, 43.1%; Table 3). Most participants rated their health status as “good to excellent” (n=775, 79.7%). Participants reported a median DM duration of 6.3 (IQR 3.0-12.0) years, and most were on diet control (n=915, 94.2%). Participants had median HbA_1c_, and systolic and diastolic blood pressure values of 6.9% (IQR 6.4%-7.6%), and 130.0 (IQR 120.0-138.0) mm Hg and 70.0 (IQR 65.0-78.0) mm Hg, respectively. The median low-density lipoprotein cholesterol and eGFR was 2.1 (IQR 1.7-2.6) mmol/L and 88.5 (IQR 73.8-98.3) mL/min/1.73m^2^, respectively.

**Table 3:** Baseline medical and clinical characteristics of UNADS^a^ study participants.

Characteristics					Values		
**Diabetes Management**							
	Diabetes duration, median (IQR)			6.3 (3.0-12.0)			
	**Diabetes treatment, n (%)**						
		Insulin		68 (7)			
		Oral antidiabetic medications		789 (81.3)			
		Diet		915 (94.2)			
		Exercise		780 (80.3)			
**Health status**							
	**Comorbidities**						
		None, n (%)		54 (5.6)			
		1-2 conditions, n (%)		640 (66.5)			
		3 conditions or more, n (%)	268 (27.9)				
		Hypertension (yes), n (%)		711 (73.4)			
		Dyslipidemia (yes), n (%)		828 (85.1)			
		Asthma (yes), n (%)		51 (5.2)			
		BMI (kg/m2)^b^, median (IQR)		26.9 (24.1-30.3)			
		Obesity (yes), n (%)		419 (43.1)			
		Arthritis (yes), n (%)		96 (9.9)			
	**Self-rated health status, n (%)**						
		Excellent or very good		281 (28.9)			
		Good		494 (50.8)			
		Fair or poor		198 (20.4)			
**Clinical factors**							
	**Blood pressure (mm Hg), median (IQR)**						
		Systolic		130.0 (120.0-138.0)			
		Diastolic		70.0 (65.0-78.0)			
	HbA_1c_^c^ (%), median (IQR)			6.9 (6.4-7.6)			
	**Lipids (mmol/L), median (IQR)**						
		Total cholesterol		4.1 (3.6-4.7)			
		HDL^d^		1.3 (1.1-1.5)			
		LDL^e^		2.1 (1.7-2.6)			
		TGL^f^		1.4 (1.0-1.8)			
	eGFR^g^ (ml/min/1.72m^2^), median (IQR)			88.5 (73.8-98.3)			

^a^UNADS: Understanding Non-Adherence to Diabetes Complications Screening.

^b^BMI was calculated as weight in kilograms divided by height in meters squared.

^c^HbA_1c_: hemoglobin A_1c_.

^d^HDL: high-density lipoprotein.

^e^LDL: low-density lipoprotein.

^f^TGL: triglyceride level.

^g^eGFR: estimated glomerular filtration rate.

### Patient-Reported

In general, most study participants (n=764, 95.0%) had adequate health literacy (Table 4). More than half (n=532, 55.1%) reported not having received DM education either at the time of diagnosis of DM or in the past year. Most reported receiving “quite a lot or a great deal” of DM support (n=678, 69.7%). The median baseline diabetes knowledge scores of those on insulin and not on insulin were 16.0 (IQR 13.0-18.0) out of 22 and 9.0 (IQR 8.0-11.0) out of 14, respectively. Over 10% and 20% of the participants on insulin and not on insulin answered less than 50% of the questionnaire correctly, respectively. Participants reported low levels of diabetes-related self-efficacy (median 1.4, IQR 1.0-3.9 out of 5), and more than half rated their diabetes control status as “okay” (n=547, 56.6%). Almost one-fifth reported diabetes related-distress (n=171, 17.6%).

**Table 4:** Baseline patient-reported characteristics of UNADS^a^ study participants.

Characteristics			Values
**Health literacy^b^, n (%)**			
	Not adequate	41 (5.1)	
	Adequate	764 (95.0)	
Health literacy (3-item)^c^, median (IQR)			10.0 (7.0-12.0)
**Diabetes knowledge^d^, median (IQR)**			9.0 (8.0-11.0)
	Not on insulin treatment	9.0 (8.0-11.0)	
	On insulin treatment	16.0 (13.0-18.0)	
Diabetes education^e^ (no), n (%)			532 (55.1)
**Diabetes support (yes), n (%)**			
	No or a little bit of support	295 (30.3)	
	Quite a lot or a great deal of support	678 (69.7)	
**Satisfaction with diabetes support, n (%)**			
	Not at all or a little satisfied	143 (14.7)	
	Somewhat satisfied	180 (18.5)	
	Very satisfied or extremely satisfied	648 (66.7)	
**Self-rated diabetes control, n (%)**			
	Very good	255 (26.4)	
	Okay	547 (56.6)	
	Not good	80 (8.3)	
	Variable	84 (8.7)	
**Attitude toward glycemic control, n (%)**			
	Very important	916 (94.2)	
	A little or not important	56 (5.8)	
Self-efficacy^f^, median (IQR)			1.4 (1.0-3.9)
Diabetes-related distress^g^ (yes), n (%)			171 (17.6)

^a^UNADS: Understanding Non-Adherence to Diabetes Complications Screening.

^b^Adequate health literacy was defined as 75% correct response in both sections, that is, 3 numeracy items and 27 reading comprehension sections.

^c^Higher score indicates higher health literacy level.

^d^Higher score indicates higher level of diabetes knowledge.

^e^Have you ever attended any diabetes education programs focusing on diabetic retinopathy, diabetic nephropathy, or diabetic foot complications management?

^f^Higher score indicates higher self-efficacy level.

^g^A total score of 8 or more suggests “possible” diabetes distress.

## Discussion

We established the UNADS study to provide urgently needed data on the rates, predictors, and clinical and economic impact of nonadherence to DM complications screening in a multiethnic Asian primary care population with T2DM using a mixed methods approach. A total of 974 patients with T2DM who completed 2123 DM complications screening visits across 6 polyclinics in Singapore were enrolled and have completed the baseline assessment. Most (n=793, 81.4%) were recruited while attending more than 1 complication screening on the same day. A spectrum of sociodemographic (eg, age, ethnicity, education and income levels, housing, and accessibility to nearest polyclinic), medical (eg, presence of comorbidities, and HbA_1c_ and lipid levels), and patient-reported factors (eg, DM-specific self-efficacy and knowledge levels, and self-rated DM control) which may potentially predict nonadherence to DM complications screening, were systematically collected. Completion of longitudinal quantitative and cross-sectional qualitative data collection and analyses will provide insights into the rates and modifiable risk factors of nonadherence to DM complications screening in this population; and contribute to the design of evidence-based interventions addressing those factors at patient, provider, and system levels in the public sector.

We found that more than two-fifths of our study participants were aged less than 60 years, were mostly Chinese, had secondary or lower education, and received a monthly household income of more than S $2000 (US $1546.05). These characteristics have been reported to be linked to nonadherence to DM complications screening by previous studies exploring factors related to attendance to DM-related screening programs [8,43]. For example, a large cohort study (n=24,832) of individuals with DM by Alice et al [8] reported that those aged less than 60 years and receiving a higher income were associated with the greatest risk (–1.3-fold) of nonadherence to eye examination guidelines in New South Wales. Furthermore, a mixed methods study investigating the barriers or facilitators of uptake of DM health screening conducted in Singapore showed that those of Chinese ethnicity (vs Indians), and those with lower education had lower odds of attending regular DM health screenings [43].

Our baseline analysis also showed a high prevalence of comorbid conditions such as hypertension, dyslipidemia, and obesity, which may be associated with an increased likelihood of not attending screening appointments. Comorbidity is common among people with DM and can create a number of challenges that affect a patient’s ability to participate in regular screening [44]. Indeed, Baumeister et al [45] studied trends of barriers to receiving recommended eye care among individuals with DM in Germany using population-based data collected between 1997-2001 and 2008-2012 and found that eye care use declined over the 10-year period in those diagnosed with DM for more than 5 years, and in those with obesity, dyslipidemia, and multiple comorbid conditions.

We found that more than half of our baseline study sample had not received any DM-related education prior to DM complication screening. This is important, as evidence suggests that a lack of information on DM may predict nonadherence to regular annual screening. For instance, in a nationwide survey of 1288 individuals with DM conducted in Korea, Byun et al [12] found that those who had not received education about DM care were more likely to be screened less often for DR and DN. At baseline, our participants reported low levels of DM-specific self-efficacy, which may be important in predicting nonadherence to DM complications screening. Indeed, studies exploring screening attendance in other health care fields (eg, cancer) have shown that low self-efficacy is an important determinant of nonadherence to screening programs [46]. Nevertheless, full analysis of the follow-up data using regression models is essential to identify and confirm the modifiable predictors of DM complications screening in this population and to inform appropriate intervention in this multiethnic primary care population.

A key strength of the UNADS study is the application of the PRECEDE-PROCEED model, which offers a systematic classification of factors at individual, provider, and system levels by their relative importance and capacity for modification. This facilitates consideration of the determinants for change at individual-, provider-, and system-levels and allows for the development of more efficient and effective programs. Other strengths are its large sample size involving six polyclinics across 2 different health care clusters and the collection of rich longitudinal quantitative and qualitative data covering a wide range of exposures and outcomes.

The UNADS study has some important limitations. First, the COVID-19 outbreak and related restrictions had serious implications on its implementation. Due to the restrictions on face-to-face research activities, study enrollment was severely impacted, and the estimated target sample size was not achieved. However, as far more of our study participants attended bundle screening (81.4%) than originally estimated in our sample size calculations (30%), our final sample size was deemed adequate to accomplish our study’s objectives. Secondly, our overall baseline response rate was low (35%) and this could lead to selection bias affecting the validity of our results. There were several reasons for this: survey fatigue experienced by patients attending busy sites like Bedok and Bukit Merah with several ongoing research projects; the need for biological sample collection at baseline and follow-up (if unavailable in the EMR); and the impact of restrictions imposed during the COVID-19 outbreak as explained above [47,48]. Third, we are unable to compare the predictors of nonadherence between 2 different complication screenings (eg, DR vs DFC, DFC vs DN) due to the small number of patients attending both DR and DFC (n=223), and DFC and DN (n=123) screenings. Finally, the use of self-reported measures may be subject to social desirability and recall bias.

The UNADS study is the first longitudinal mixed methods study to investigate the rates, independent predictors, and the clinical and economic impact of nonadherence to DM complications screening in multiethnic polyclinic patients with T2DM in Singapore. Our baseline data are comprehensive including several sociodemographic, clinical, health, and patient-reported characteristics that may predict nonadherence to DM complications screening. Completion of the follow-up phase will provide empirical evidence on the rates, and modifiable barriers or enablers of nonadherence to DM complications screening. Furthermore, the study results may inform public health interventions to develop culturally appropriate intervention programs to improve screening adherence in multiethnic populations. Finally, this study may contribute to reducing unnecessary disease burden with timely screening, monitoring, and treatment; improving health outcomes for Singaporeans with T2DM at the primary care level; and demonstrating significant cost-savings to the patient and society over time.

## Appendix

Multimedia Appendix 1 Supplementary Figures.

Multimedia Appendix 2 Details of the UNADS study procedures, participants and timeline for administration.

Multimedia Appendix 3 Consolidated Criteria for Reporting Qualitative Research (COREQ) checklist.

Multimedia Appendix 4 Additional materials.

## Data Availability

The datasets generated and analyzed as part of this project will be available from the corresponding author on reasonable request after the research team has completed analyses related to the study aims.

## References

[1] Harding JL, Pavkov ME, Magliano DJ, Shaw JE, Gregg EW (2019). Global trends in diabetes complications: a review of current evidence. Diabetologia.

[2] Tan KW, Dickens BSL, Cook AR (2020). Projected burden of type 2 diabetes mellitus-related complications in Singapore until 2050: a Bayesian evidence synthesis. BMJ Open Diabetes Res Care.

[3] Agardh E, Agardh C, Hansson-Lundblad C (1993). The five-year incidence of blindness after introducing a screening programme for early detection of treatable diabetic retinopathy. Diabet Med.

[4] Ang GY, Yap CW, Saxena N (2017). Effectiveness of diabetes foot screening in primary care in preventing lower extremity amputations. Ann Acad Med Singap.

[5] Delevry D, Ho A, Le QA (2021). Association between processes of diabetes care and health care utilization in patients with diabetes: evidence from a nationally representative US sample. J Diabetes.

[6] Imai C, Li L, Hardie R, Georgiou A (2021). Adherence to guideline-recommended HbA1c testing frequency and better outcomes in patients with type 2 diabetes: a 5-year retrospective cohort study in Australian general practice. BMJ Qual Saf.

[7] Palmer AJ, Valentine WJ, Chen R, Mehin N, Gabriel S, Bregman B, Rodby RA (2008). A health economic analysis of screening and optimal treatment of nephropathy in patients with type 2 diabetes and hypertension in the USA. Nephrol Dial Transplant.

[8] Gibson AA, Humphries J, Gillies M, Nassar N, Colagiuri S (2020). Adherence to eye examination guidelines among individuals with diabetes: an analysis of linked health data. Clin Exp Ophthalmol.

[9] Rim THT, Byun IH, Kim HS, Lee SY, Yoon JS (2013). Factors associated with diabetic retinopathy and nephropathy screening in Korea: the Third and Fourth Korea National Health and Nutrition Examination Survey (KNHANES III and IV). J Korean Med Sci.

[10] Tapp RJ, Zimmet PZ, Harper CA, de Courten MP, Balkau B, McCarty DJ, Taylor HR, Welborn TA, Shaw JE (2004). Diabetes care in an Australian population: frequency of screening examinations for eye and foot complications of diabetes. Diabetes Care.

[11] Huang OS, Tay WT, Ong PG, Sabanayagam C, Cheng C, Tan GS, Cheung GCM, Lamoureux EL, Wong TY (2015). Prevalence and determinants of undiagnosed diabetic retinopathy and vision-threatening retinopathy in a multiethnic Asian cohort: the Singapore Epidemiology of Eye Diseases (SEED) study. Br J Ophthalmol.

[12] Byun S, Ma SH, Jun JK, Jung K, Park B (2013). Screening for diabetic retinopathy and nephropathy in patients with diabetes: a nationwide survey in Korea. PLoS One.

[13] Eppley SE, Mansberger SL, Ramanathan S, Lowry EA (2019). Characteristics associated with adherence to annual dilated eye examinations among US patients with diagnosed diabetes. Ophthalmology.

[14] Fayfman M, Schechter MC, Amobi CN, Williams RN, Hillman JL, Alam MM, Rajani RR, Ziemer DC, Kempker RR, Umpierrez GE (2020). Barriers to diabetic foot care in a disadvantaged population: a qualitative assessment. J Diabetes Complications.

[15] Hipwell AE, Sturt J, Lindenmeyer A, Stratton I, Gadsby R, O'Hare P, Scanlon PH (2014). Attitudes, access and anguish: a qualitative interview study of staff and patients' experiences of diabetic retinopathy screening. BMJ Open.

[16] Hung SLL, Fu SN, Lau PS, Wong SYS (2015). A qualitative study on why did the poorly-educated Chinese elderly fail to attend nurse-led case manager clinic and how to facilitate their attendance. Int J Equity Health.

[17] van Eijk KND, Blom JW, Gussekloo J, Polak BCP, Groeneveld Y (2012). Diabetic retinopathy screening in patients with diabetes mellitus in primary care: incentives and barriers to screening attendance. Diabetes Res Clin Pract.

[18] Sachdeva A, Stratton I, Unwin J, Moreton R, Scanlon P (2012). Diabetic retinopathy screening: Study to determine risk factors for non-attendance. Diabetes Prim Care.

[19] Garreta Rufas A, Meredith K, Harris J, Rossing P, Hobbs R, Wanner C (2022). MO369: A systematic review to evaluate the drivers of non-adherence to albuminuria testing guidelines and the clinical and economic impact of not identifying CKD over the course of progressive kidney function loss. Nephrology Dialysis Transplantation.

[20] Kashim R, Newton P, Ojo O (2018). Diabetic retinopathy screening: A systematic review on patients' non-attendance. Int J Environ Res Public Health.

[21] Leese GP, Boyle P, Feng Z, Emslie-Smith A, Ellis JD (2008). Screening uptake in a well-established diabetic retinopathy screening program: the role of geographical access and deprivation. Diabetes Care.

[22] Green L, Kreuter M (2005). Health Program Planning: An Educational and Ecological Approach.

[23] Saulle R, Sinopoli A, De Paula Baer A, Mannocci A, Marino M, De Belvis AG, Federici A, La Torre G (2020). The PRECEDE-PROCEED model as a tool in public health screening: a systematic review. Clin Ter.

[24] Studts CR, Tarasenko YN, Schoenberg NE (2013). Barriers to cervical cancer screening among middle-aged and older rural appalachian women. J Community Health.

[25] Tramm R, McCarthy A, Yates P (2012). Using the precede-proceed model of health program planning in breast cancer nursing research. J Adv Nurs.

[26] Unger-Saldaña K, Saldaña-Tellez M, Potter MB, Van Loon K, Allen-Leigh B, Lajous M (2020). Barriers and facilitators for colorectal cancer screening in a low-income urban community in Mexico City. Implement Sci Commun.

[27] Toh M, Lee L, Tham L (2014). Quality care of diabetes mellitus in the Singapore national healthcare group: 5-year trend from 2008 to 2012.

[28] Brooke P, Bullock R (1999). Validation of a 6 item cognitive impairment test with a view to primary care usage. Int J Geriat Psychiatry.

[29] Manolio TA, Fishel SC, Beattie C, Torres J, Christopherson R, Merritt WT, Whelton PK (1988). Evaluation of the dinamap continuous blood pressure monitor. Am J Hypertens.

[30] Levey AS, Stevens LA, Schmid CH, Zhang Y, Castro AF, Feldman HI, Kusek JW, Eggers P, Van Lente F, Greene T, Coresh J (2009). A new equation to estimate glomerular filtration rate. Ann Intern Med.

[31] Fitzgerald JT, Funnell MM, Anderson RM, Nwankwo R, Stansfield RB, Piatt GA (2016). Validation of the revised brief Diabetes Knowledge Test (DKT2). Diabetes Educ.

[32] Zainudin S, Ang D, Soh A (2017). Knowledge of diabetes mellitus and safe practices during Ramadan fasting among Muslim patients with diabetes mellitus in Singapore. Singapore Med J.

[33] Ko Y, Lee JY, Toh MPHS, Tang W, Tan AS (2012). Development and validation of a general health literacy test in Singapore. Health Promot Int.

[34] Chew LD, Bradley KA, Boyko EJ (2004). Brief questions to identify patients with inadequate health literacy. Fam Med.

[35] Anderson RM, Fitzgerald JT, Gruppen LD, Funnell MM, Oh MS (2003). The Diabetes Empowerment Scale-Short Form (DES-SF). Diabetes Care.

[36] McGuire BE, Morrison TG, Hermanns N, Skovlund S, Eldrup E, Gagliardino J, Kokoszka A, Matthews D, Pibernik-Okanović M, Rodríguez-Saldaña J, de Wit M, Snoek FJ (2010). Short-form measures of diabetes-related emotional distress: the Problem Areas in Diabetes Scale (PAID)-5 and PAID-1. Diabetologia.

[37] Speight J, Browne JL, Holmes-Truscott E, Hendrieckx C, Pouwer F (2012). Diabetes MILES—Australia (management and impact for long-term empowerment and success): methods and sample characteristics of a national survey of the psychological aspects of living with type 1 or type 2 diabetes in Australian adults. BMC Public Health.

[38] Tang TS, Brown MB, Funnell MM, Anderson RM (2008). Social support, quality of life, and self-care behaviors among African Americans with type 2 diabetes. Diabetes Educ.

[39] Tong A, Sainsbury P, Craig J (2007). Consolidated Criteria for Reporting Qualitative Research (COREQ): A 32-item checklist for interviews and focus groups. Int J Qual Health Care.

[40] Michie S, Johnston M, Abraham C, Lawton R, Parker D, Walker A, "Psychological Theory" Group (2005). Making psychological theory useful for implementing evidence based practice: a consensus approach. Qual Saf Health Care.

[41] French SD, Green SE, O'Connor DA, McKenzie JE, Francis JJ, Michie S, Buchbinder R, Schattner P, Spike N, Grimshaw JM (2012). Developing theory-informed behaviour change interventions to implement evidence into practice: a systematic approach using the theoretical domains framework. Implement Sci.

[42] Rice P, Ezzy D (1999). Qualitative Research Methods: A Health Focus.

[43] AshaRani PV, Devi F, Wang P, Abdin E, Zhang Y, Roystonn K, Jeyagurunathan A, Subramaniam M (2022). Factors influencing uptake of diabetes health screening: a mixed methods study in Asian population. BMC Public Health.

[44] Piette JD, Kerr EA (2006). The impact of comorbid chronic conditions on diabetes care. Diabetes Care.

[45] Baumeister SE, Schomerus G, Andersen RM, Tost F, Markus MRP, Völzke H, Jürgens C (2015). Trends of barriers to eye care among adults with diagnosed diabetes in Germany, 1997-2012. Nutr Metab Cardiovasc Dis.

[46] Bongaerts TH, Büchner FL, Middelkoop BJ, Guicherit OR, Numans ME (2020). Determinants of (non-)attendance at the Dutch cancer screening programmes: a systematic review. J Med Screen.

[47] Bixo L, Cunningham JL, Ekselius L, Öster C, Ramklint M (2021). 'Sick and tired': Patients reported reasons for not participating in clinical psychiatric research. Health Expect.

[48] Villarosa AR, Ramjan LM, Maneze D, George A (2021). Conducting population health research during the COVID-19 pandemic: impacts and recommendations. Sustainability.

