# Supplementary material for: Nonadherence to Diabetes Complications Screening in a Multiethnic Asian Population: Protocol for a Mixed Methods Prospective Study
Source: JMIR Res Protoc. 2025 May 8;14:e63253. doi: 10.2196/63253 (PMC12099272; doi:10.2196/63253)
Supplement: Multimedia Appendix 1 [file resprot_v14i1e63253_app1.docx]

**Supplementary Figure 1. Application of PRECEDE component in the UNADS study**

**Phases of the PRECEDE Component**

1. Social Assessment

2. Epidemiological Assessment

3. Behavioural and Environmental Assessment

4. Educational and Ecological Assessment

Predisposing factors (Knowledge, attitudes, beliefs, perceptions)

Enabling factors (availability of resources, accessibility, skills)

Reinforcing factors (attitudes and behaviours of peers, family, mass media)

5. Administrative and Policy Assessment

**Data Sources/ Methods Employed**

**Qualitative:** Key informant interviews with stakeholders in Polyclinics

**Quantitative:** Ministry of Health Primary Care Survey 2010, SEED Population based cohort

**Quantitative:** clinical and ocular data collection and questionnaire administration

**Qualitative:** Focus groups/semi-structured interviews with patients and health care professionals

**Qualitative:** Key Informant Interviews with stakeholders in polyclinics

**Identification of the Health problem**

**Setting Behavioural and Environmental Objectives**

**Setting Sub objectives**

**Setting strategy objectives**

Theoretical Domains Framework

Identification of modifiable factors that would result in behaviour change

Identification of key environmental and behavioural factors related to the health problem

Investigation of the influence of organizational resources and policies

**Supplementary Figure 2. UNADS Study Screening and Recruitment**

DM Complication Screening at SingHealth Polyclinic

SHP Bedok

n= 1038

Eligible n=2716

Ineligible n=830

SHP Outram

n= 639

SHP Pasir Ris

n= 410

SHP Bukit Merah

n= 552

Total screened

N=3546

Agreed n=974 (35.9%)

Refused n=1742 (64.1%)

Received <12 months rescreening referral n=604 (72.8%)

Referral to tertiary care n=39 (4.7%)

Significant hearing Impairment n=63 (7.6%)

Cognitive impairment n=38 (4.6%)

Not Chinese/Malay/Indian/

Eurasian n=35 (4.2%)

Others* n=51 (6.1%)

DM Complication Screening at National Healthcare Group Polyclinic

NHGP Hougang

n= 512

NHGP Geylang

n= 395

Others* = Not Singaporean/Singapore permanent resident, type 1 diabetes, less than 21 years, residing at nursing home, not English/Mandarin/Malay/Tamil speaker
