# Supplementary material for: Nonadherence to Diabetes Complications Screening in a Multiethnic Asian Population: Protocol for a Mixed Methods Prospective Study
Source: JMIR Res Protoc. 2025 May 8;14:e63253. doi: 10.2196/63253 (PMC12099272; doi:10.2196/63253)
Supplement: Multimedia Appendix 2 [file resprot_v14i1e63253_app2.docx]

| **Supplementary Table 1.** Details of the UNADS study procedures, participants and timeline for administration | | | | | |
| --- | --- | --- | --- | --- | --- |
| **Construct** | **Time Administered** | | **Participant type** | **Assessment tool/device** | **Description** |
|  | **Baseline** | **Follow-up** |  |  |  |
| Sociodemographic characteristics | **•** |  | Patients | Polyclinic medical records and in-house questionnaire | Age, gender, race, marital status, highest education level, occupation, monthly household income, language spoken at home, housing type, religion, and living arrangements |
| Sociodemographic characteristics |  | **•** | Healthcare professionals | In-house questionnaire | Age, gender, race, nationality, marital status, language spoken, religion, highest education level, current designation, number of years work history in the current position, department, institution, number of years work history as a healthcare provider |
| Lifestyle/social History | **•** |  | Patients | Polyclinic medical records and in-house questionnaire | Smoking and alcohol consumption |
| Diabetes and related complications history, and other comorbidities | **•** |  | Patients | Polyclinic medical records and in-house questionnaire | Duration of DM, history of DM related complications and other existing medical conditions, current DM treatment, utilization of education programs focusing on DM complications management |
| Anthropometry | **•** |  | Patients | Polyclinic medical records / measurement on the day of assessment   - Height - Wall-mounted measuring tape - Weight - Calibrated digital scientific weight scale (SECA, model 782 2321009; Vogel & Halke, Germany) | Height was measured in cm  Weight was measured in kg  Body mass index (BMI) was calculated as weight in kilograms divided by height in meters squared (Wt[kg]/Ht[m]2) and was categorized as underweight (<18.5 kg/m2), normal (18.5-23 kg/m2), overweight (23-27.5 kg/m2) and obese (> 27.5 kg/m2) according to Asian cut-offs. (1) |
| Blood and Urine Parameters | **•** | **•** | Patients | Polyclinic medical records /collection of venous blood & urine samples  Blood and urine samples were processed and analysed at Innoquest Laboratories Pte Ltd (SHP) or National Healthcare Diagnostics (NHGP) in Singapore for measurement on the same day. | Venepuncture was conducted by the polyclinic nurses.  Blood tests- glycated Haemoglobin (HbA1C), lipids (total cholesterol, high density lipoprotein cholesterol (HDL) and low density lipoprotein cholesterol (LDL), and triglycerides), and serum creatinine  Urine tests – eGFR and urine albumin/Creatinine ratio |
| Blood pressure (BP; systolic & diastolic) | **•** | **•** | Patients | Polyclinic medical records/ collected on the day of assessment using digital automatic blood pressure monitor(2) (Dinamap model Pro Series DP110X-RW, 100V2; GE Medical Systems Information Technologies, Inc., USA) | BP was taken with the participant seated and after 5 minutes of rest. It was measured on two occasions, 5 minutes apart. If the readings differed by more than 10 mmHg (systolic) or 5 mmHg (diastolic), a third reading was taken. The BP of the individual was then taken as the mean between the two closest readings. |
| Ocular Parameters | **•** | **•** | Patients | Polyclinic SiDRP records | DR diagnosis (Only for participants attending DR screening)  Category – None (ETDRS Level 10; Normal fundus, any flame haemorrhage (FH), cotton wool spots in the absence of dot haemorrhage (DH) /Micro aneurysms (MA), any hard exudates (HE) in the absence of DH/MA outside of outer zone)  Category – Mild non-proliferative DR (ETDRS Level 20-35; One or more of the following – DH/MA, 1 or 2 blot haemorrhage, HE in the absence of DH/MA within macula zone |
| Diabetes-related Knowledge | **•** |  | Patients | Modified 22-item version of the validated Revised Diabetes Knowledge Test (DKT) (3, 4) | The DKT was used to evaluate and measure the diabetes related knowledge as the outcome. All of the questions from the original Revised DKT were retained, except for question 5, and one option each in questions 1,2, 3 and 8 were modified to better suit the Singapore population. Furthermore, question 4 was removed. This modified 22-item test contains two segments – 1.a general segment comprising 14 items appropriate for patients with T2DM; 2. an optional segment comprising 8 items and is only appropriate for T2DM patients using insulin. Participants were required to choose the best answer for each of the multiple choice items. Each segment was scored separately, and the maximum possible score was 14 or 22 (if the participant was on insulin treatment). |
| Health Literacy | **•** |  | Patients | Health literacy test for Singapore (HLTS)(5) | Health literacy for DM was assessed using the HLTS developed and culturally adapted from the Short Test of Functional Health Literacy for Adults (STOFHLA). The HLTS contains a 4-item numeracy test and a 36-item reading comprehension test that consists of two prose passages. Adequate HL was defined as 75% correct responses in both HLTS sections (i.e.,> 3 numeracy items and 27 reading comprehension items) |
| Health Literacy | **•** |  | Patients | 3 items from STOFHLA (6) | Three health literacy screening questions from the STOFHLA (16 items questionnaire) were used to identify patients with inadequate or marginal health literacy. Each item is rated on a five-point Likert scale ranging from ‘Never/Not at all’ to ‘Always/Extremely’. The health literacy score is calculated by summing the 3 item scores (0-12). |
| Self-Efficacy | **•** |  | Patients | Diabetes Empowerment Scale – Short Form (DES-SF) (7) | The DES-SF was used to measure the psychosocial self-efficacy of patients with diabetes. It measures patients’ self-efficacy related to managing the psychosocial aspects of diabetes, assessing dissatisfaction and readiness to change, and setting and achieving diabetes goals using 8 items. Each item is rated on five-point Likert scale ranging from ‘Strongly agree (5)’ to ‘strongly disagree (1)’. A higher score indicates higher self-efficacy. |
| Diabetes-related distress | **•** |  | Patients | Problem Areas in Diabetes Scale (PAID-5)(8) | The PAID-5 was used to identify diabetes-related distress in patients with diabetes. It contains 5 items which have a five-point rating scale (0–4 representing ‘Not a problem’ through to ‘Serious problem’). Scores ranged between 0 and 20 with higher scores indicating greater diabetes-related distress and a total score > 8 suggests ‘possible’ diabetes distress. |
| Diabetes-related social support | **•** |  | Patients | Questions related to social support (9, 10) | Two items inspired by Tang et al(10) were used to assess total amount of social support received (whether positive or negative) and satisfaction with support received. The responses were rated on 4- and 5-point Likert-type scales from ‘no support’ to ‘a great deal of support’ and ‘not at all satisfied’ to ‘extremely satisfied’, respectively. |
| History of adherence/non-adherence to screening | **•** |  | Patients | In-house questionnaire | Since being diagnosed with diabetes, how often have you attended screening for DR/DN/DFC? Each item has five options – ‘once every year’, ‘once every two years’, ‘rarely’, ‘never’, and ‘unsure’. |
| Adherence/non-adherence to DM management | **•** |  | Patients | In-house questionnaire | - On average, how many times a week do you miss taking your diabetes medications? - On average, how many times a year do you run out of your medications for at least a day or two? |
| Time taken to reach screening appointments | **•** |  | Patients | In-house questionnaire | How long does it take to go to the nearest Polyclinic for your annual DM complications screening? This item has four options – ‘<30 minutes’, ’30 minutes to 1 hour’, ‘one to two hours’, and ‘don’t know’. |
| Diabetes and diabetes complications related education needs and experiences | **•** |  | Patients | In-house questionnaire | - Have you ever attended any diabetes education programs focusing on DR/DN/DFC management? This item had four options – ‘no’, ‘yes, in the past year’, ‘yes, at diagnosis of diabetes or soon after’, and ‘unsure’. - What kind of diabetes education have you received? This item had three options – ‘one-to-one with diabetes educator’, ‘group education’, and ‘internet-based education program’. - If you have not attended a structured diabetes education program focusing on DR/DN/DFC, why is that? Multiple choice options were provided including ‘not available in my area’, ‘distance is a problem’, ‘I did not have the time’ were’, and ‘unsure’. |
| Attitudes towards Diabetes control | **•** |  | Patients | In-house questionnaire | - How important is blood sugar control to your health? The responses were rated on 3-item Likert scale - ‘1= very important’ to ‘3=not important’ were given. - In the last 12 months, how would you rate your diabetes control? The responses were rated on 3-item scale – ‘1= very good’ to ‘3=not good’. - How many days a week do you measure your blood sugar levels at home? Seven options were provided including ‘never’, ‘less than once a week’,’1-2 days a week’, ‘3-4 days a week’, ‘5-6 days a week’, ‘7 days a week’, and ‘variable’. - What prevents you from checking your blood sugar levels at home? Multiple choice options were provided including ‘my diabetes is not that severe’ to ‘my physical conditions prevent me’, ‘others’ and ‘don’t know’. |
| Adherence/non-adherence to DM complications screening |  | **•** | Patients | Polyclinic Medical records | Scheduled date of 12-month follow-up visit, and actual date of attendance |
| Barriers and facilitators to DM complications screening guidelines |  | **•** | Patients and Healthcare professionals | In-house interviewers guide | Focus groups /semi-structured interviews on adherence/non-adherence to recommended DM complications screening guidelines with patients and healthcare professionals based on the PRECEED and Theoretical Domains Framework, respectively. |

**REFERENCES**

1. Tan K. Appropriate body-mass index for Asian populations and its implications for policy and intervention strategies. The lancet. 2004.

2. Manolio TA, Fishel SC, Beattie C, Torres J, Christopherson R, Merritt WT, et al. Evaluation of the Dinamap continuous blood pressure monitor. American journal of hypertension. 1988;1(3 Pt 3):161s-7s.

3. Fitzgerald JT, Funnell MM, Anderson RM, Nwankwo R, Stansfield RB, Piatt GA. Validation of the Revised Brief Diabetes Knowledge Test (DKT2). Diabetes Educ. 2016;42(2):178-87.

4. Zainudin SB, Ang DY, Soh AW. Knowledge of diabetes mellitus and safe practices during Ramadan fasting among Muslim patients with diabetes mellitus in Singapore. Singapore Med J. 2017;58(5):246-52.

5. Ko Y, Lee JY, Toh MP, Tang WE, Tan AS. Development and validation of a general health literacy test in Singapore. Health Promot Int. 2012;27(1):45-51.

6. Chew LD, Bradley KA, Boyko EJ. Brief questions to identify patients with inadequate health literacy. Fam Med. 2004;36(8):588-94.

7. Anderson RM, Fitzgerald JT, Gruppen LD, Funnell MM, Oh MS. The Diabetes Empowerment Scale-Short Form (DES-SF). Diabetes Care. 2003;26(5):1641-2.

8. McGuire BE, Morrison TG, Hermanns N, Skovlund S, Eldrup E, Gagliardino J, et al. Short-form measures of diabetes-related emotional distress: the Problem Areas in Diabetes Scale (PAID)-5 and PAID-1. Diabetologia. 2010;53(1):66-9.

9. Speight J, Browne JL, Holmes-Truscott E, Hendrieckx C, Pouwer F. Diabetes MILES--Australia (management and impact for long-term empowerment and success): methods and sample characteristics of a national survey of the psychological aspects of living with type 1 or type 2 diabetes in Australian adults. BMC Public Health. 2012;12:120.

10. Tang TS, Brown MB, Funnell MM, Anderson RM. Social support, quality of life, and self-care behaviors amongAfrican Americans with type 2 diabetes. Diabetes Educ. 2008;34(2):266-76.
