# Supplementary material for: Nonadherence to Diabetes Complications Screening in a Multiethnic Asian Population: Protocol for a Mixed Methods Prospective Study
Source: JMIR Res Protoc. 2025 May 8;14:e63253. doi: 10.2196/63253 (PMC12099272; doi:10.2196/63253)
Supplement: Multimedia Appendix 4 [file resprot_v14i1e63253_app4.pdf]

PROCEDE-PROCEED Model

The PRECEDE-PROCEED Model has two distinct components: 1. 'PRECEDE' (Predisposing, Reinforcing and Enabling Constructs in Educational Diagnosis and Evaluation), which constitutes five diagnostic and planning phases to facilitate the identification of priorities and setting of objectives; and 2. PROCEED (Policy, Regulatory, and Organizational Constructs in Educational and Environmental Development), which consists of four phases and focuses on identification of criteria for policy implementation and subsequent evaluation. In UNADS, we only utilized the PRECEDE aspect of the model. The first phase (social diagnosis) establishes the health problems which impact the quality of life of the target population and the second phase (epidemiological diagnosis) documents the importance of these problems through survey and epidemiological data. The third phase (behavioural/environmental diagnosis) focuses on the systematic identification of health practices and other factors linked to the health problems. The fourth phase (educational and ecological diagnosis) identifies the factors which, when modified, will most likely result in the desired behaviour change. These factors are then classified as predisposing, enabling, or reinforcing. The last phase (administrative/ policy assessment) of PRECEDE analyses policies, resources and circumstances prevailing organizational situations that could hinder or facilitate meeting the objectives of phase 4.

Data storage

Quantitative data

Clinical and ocular records, and questionnaire responses were compiled into participant-specific case report forms that were labelled with the participant's unique study number. Hard copies of the data collected were stored in a secure cabinet at each study site. Identifiable data were password-protected and stored in shared drives at each study site. Every quarter, data were extracted and checked for quality issues by the Data Management Unit at the Singapore Eye Research Institute (SERI).

Qualitative data

Audio-recordings and transcripts were stored in password-protected folders in shared drives of SERI.

A guide to interview adherent and non-adherent UNADS participants

This is a tentative guide and can be edited where applicable

Introduction and purpose

Many thanks for taking the time to join this focus group. My name is XXXX. I am a research (fellow/assistant) at the Singapore Eye Research Institute and, today, I will be the 'moderator' for this group discussion. This is my colleague YYYY and he/she will be the 'note-taker' for this session.

You were chosen to attend this focus group because you had participated in a study called Understanding Non-adherence to Diabetes Complications Screening, or in short called UNADS, during your visit to the Polyclinic approximately 12 months ago. The study aims to identify and understand the reasons why patients choose to attend or not to attend their Polyclinic screening appointments for their diabetes complications from a patient's and healthcare provider's perspective. This important information will help us create guidelines to increase the rate of attendance.

Today, in this group discussion, we are interested in finding out the reasons why people attend or not attend their Polyclinic screening appointments for diabetes complications such as diabetic retinopathy or diabetic eye disease, diabetic neuropathy or diabetic foot disease, and diabetic nephropathy or diabetic kidney disease. We would like to hear your views, and share your experiences and opinions related to this.

Focus group guidelines

My role here is to ask questions and listen. I won't actually be participating in the conversation, instead I would like you to feel free to talk to one another and have discussions in response to the questions. I will be asking questions and moving the discussion from one topic to the next. If you have any specific questions to ask me, we can cover those at the end of the focus group.

I would like to stress that there are **no right or wrong answers** and we are most interested in your personal opinions and experiences. I would really like to encourage you to share your point of view, even if it differs from what others have said. We are not looking to have everyone agree, so if you find that you are having a different opinion to the rest of the group I would really like to hear it.

Often in groups some people talk a lot and others say little. It is important for us to hear from everyone, as you all have different experiences. So if someone is sharing a lot, I may ask you to let others talk, and if you aren't saying much, I may ask you for your opinion. Also, as we have limited time, I may need to interrupt and redirect our discussion if it is going off on a tangent.

You don't need to speak in any particular order, please feel free to speak at any time, but try not to speak when someone else is talking.

We're recording the session because we don't want to miss any of your comments. Whilst you may mention names in your discussion, please be assured that your names will not be reported in any later reports or publications on this work. Everything will remain confidential. In order to protect other's privacy, we request you to not discuss details outside the group

Today's focus group session will last for about an hour.

Does anyone have any questions so far?

*****Moderator/Interviewer: Turn on the recorder now*****

Warm up questions

Let's start by getting to know each other. Let's go around the group. Briefly, please let us know your name and how long you have had diabetes.

Questions and prompts

I'll now start asking some general questions and then more specific questions as we go along.

The following questions were used to guide the interviews with participants who were adherent to diabetes mellitus complications screening at the 12-month follow-up:

<u>Enabling Factors</u>

Can you tell me why you chose to attend your screening appointment at the Polyclinic?

What were some of the reasons you chose to attend?

What things made it easy for you to attend your screening appointment?

What things influenced your decision to attend your screening appointment?

What things are troublesome about having to attend your diabetes screening appointment?

What aspects of your screening appointments make you feel like you don't want to attend them? (e.g., pain from drops, waiting times, inconvenience etc.).

Why didn't these things stop you from attending?

What things did you need to organise so that you could attend your screening appointment?

Was this easy or difficult? Why?

What would encourage you to keep attending your screening appointments in the future?

Is there anything that would make it easier for you to attend future screening appointments?

<u>Predisposing Factors</u>

In your own words, can you explain what diabetic eye/foot/kidney disease is?

How does diabetic eye/foot/kidney disease affect people with diabetes?

From your understanding, why do people with diabetes need to go for eye/kidney/feet checks every year?

What is the purpose of screening for diabetes complications such as eye/kidney/foot?

What have you been told about the benefits of screening for diabetes complications?

Why is it necessary?

Why do you think going for eye/foot/kidney screening is important or not important?

If you think screening is important, why is this?

If you think screening is not very important, why is this?

How do your traditional beliefs or practices (e.g. TCM) influence your decision to attend eye/foot/kidney screening at the Polyclinics?

What traditional beliefs/practices (e.g. non-Western medicine) influence your decision?

Reinforcing factors

What support do you have from your family or friends to attend your diabetes complications screening appointments?

How could they support you more?

What is your family's opinion about your appointments for diabetes complications screening?

What do they understand about your need to attend diabetes complications screening?

How do your family react to you needing to go to screening appointments for diabetes complications?

What information have you been given by your doctors about your diabetes complications screening (e.g. why it's important, why you need to go, the goal of the screening etc.)?

Who from your health care team, if anyone, has recommended that you attend a screening appointment?

How satisfied are you with the level of information about your complications screening that has been provided to you? What has been missing?

What issues have you faced in talking to your doctor about the complications screening process?

How easy is it to ask questions to your doctor?

How has your relationship with your doctors affected your decision to attend your screening appointment?

The following questions were used to guide the interviews with participants who were non-adherent to diabetes mellitus complications screening at the 12-month follow-up:

Enabling Factors

Can you tell me why you didn't attend your screening appointment at the Polyclinic?

What were some of the reasons you decided not to attend?

What things made it difficult for you to attend your screening appointment?

What things influenced your decision not to attend your screening appointment?

What things need to be overcome for you to attend your screening appointment?

What things are troublesome about having to attend your diabetes screening appointment?

What things don't you like about your screening appointments?

What aspects of your screening appointments make you feel like you don't want to attend them? (e.g. pain from drops, waiting times, inconvenience etc).

What would help you to attend your screening appointments in the future?

Is there anything that would make it easier for you to attend future screening appointments?

Predisposing Factors

In your own words, can you explain what diabetic eye/foot/kidney disease is?

How does diabetic eye/foot/kidney disease affect people with diabetes?

From your understanding, why do people with diabetes need to go for eye/kidney/feet checks every year?

What is the purpose of screening for diabetes complications such as eye/kidney/foot?

What have you been told about screening for diabetes complications?

Why is it necessary?

Why do you think going for eye/foot/kidney screening is important or not important?

If you think screening is important, why is this?

If you think screening is not very important, why is this?

How do your traditional beliefs or practices (e.g. TCM) influence your decision to not attend eye/foot/kidney screening at the Polyclinics?

What traditional beliefs/practices (i.e. non-Western medicine) that influence your decision?

Reinforcing factors

What support do you have from your family or friends to attend your diabetes complications screening appointments?

How could they support you more?

What is your family's opinion about your appointments for diabetes complications screening?

What do they understand about your need to attend diabetes complications screening?

How do your family react to you needing to go to screening appointments for diabetes complications?

What information have you been given by your doctors about your diabetes complications screening (e.g. why it's important, why you need to go, the goal of the screening etc.)?

Who from your health care team, if anyone, has recommended that you attend a screening appointment?

How satisfied are you with the level of information about your complications screening that has been provided to you? What has been missing?

What issues have you faced in talking to your doctor about the complications screening process?

How easy is it to ask questions to your doctor?

How has your relationship with your doctors affected your decision to not attend your screening appointment?

Guide for interviewing healthcare professionals

<u>Introduction</u>

Many thanks for taking the time to talk to me today. My name is XXXX and I am a research assistant at the Singapore Eye Research Institute. We are interested in finding out the reasons why people with diabetes attend or don't attend their screening appointments for diabetes complications such as diabetic retinopathy, diabetic neuropathy and diabetic nephropathy. We are really interested in hearing your views, experiences and opinions. This will help us to better understand the reasons why people choose to attend or not to attend their screening appointments for their diabetes and to help us create guidelines to increase the rate of attendance.

<u>Interview guidelines</u>

My role here is to ask questions and listen. I would like to stress that there are **no right or wrong answers** and we are most interested in your personal opinions, perceptions and experiences.

We're recording the session because we don't want to miss any of your comments. Whilst you may mention names in your discussion, these will not be reported. In any later reports about this work there will not be any names attached to comments. Everything will remain confidential. Do you have any questions so far?

*****Moderator: Turn on the recorder now*****

Questions and prompts

As I said, we are interested today in getting a better understanding about why people **attend and don't attend** their screening appointments for diabetic eye/foot/kidney. I'll start by asking some questions starting generally and then becoming more specific as we go along (**Don't read the section headings out**).

Factors influencing patients' non-adherence to screening

What are the current screening guidelines and referral practices followed in your clinic?

What factors do you think patients consider when they are deciding whether or not to attend their screening appointments?

What things make it less likely that patients will attend their screening appointments?

Why do you think some people attend their screening appointments and some don't?

What aspects of the polyclinic system do you think influences patients' decision to attend or not attend their screening appointments?

PROMPT IF NEEDED: e.g. appointment scheduling, time needed, location, travel…

How do you think the relationship between the health care team and the patient affects patients' decisions to attend or not attend their screening appointment?

PROMPT IF NEEDED: e.g. good communication, rapport, trust etc.

What information do you provide to patients regarding the referral for DR/DN/DFC screening during the consultation with them?

How do you think this information influences their decision to attend or not attend their screening appointment?

Facilitators to screening appointment adherence

In your opinion, what things make it more likely that patients will attend their screening appointments?

What factors help patients to attend their appointments?

What could be done to increase the rate of adherence to screening appointments?

How could we improve patients' adherence to DR/DN/DFC screening?

PROMPT IF NEEDED (system facilitators): e.g. better scheduling, bundle screening, less waiting times, transport assistance, awareness campaigns by government about importance of screening …

PROMPT IF NEEDED (physician facilitators): e.g. more education by healthcare staff on the value of screening, better patient-doctor communication…

PROMPT IF NEEDED (patient facilitators): better personal organisation, health literacy…
